# Effective strategy to cope the pain and discomfort among women undergoing mammography - A randomized controlled trial

**DOI:** 10.12669/pjms.39.5.7500

**Published:** 2023

**Authors:** Anila Rahim, Binish Rasheed, Syed Omair Adil, Nasreen Naz, Neha Aslam

**Affiliations:** 1 Anila Rahim, MCPS, MD. Dow Institute of Radiology, Dow University of Health Sciences, Karachi, Pakistan; 2 Binish Rasheed, FCPS. Dow Institute of Radiology, Dow University of Health Sciences, Karachi, Pakistan; 3 Syed Omair Adil, MS. School of Public Health, Dow University of Health Sciences, Karachi, Pakistan; 4 Nasreen Naz, FCPS. Dow Institute of Radiology, Dow University of Health Sciences, Karachi, Pakistan; 5 Neha Aslam, BS. Dow Institute of Radiology, Dow University of Health Sciences, Karachi, Pakistan

**Keywords:** Mammogram, Paracetamol, Pain

## Abstract

**Objectives:**

To evaluate the role of paracetamol in reducing pain and discomfort during the mammography procedure.

**Methods:**

This randomized double-blind placebo-controlled trial was conducted at DIR, Ojha and LEJ Campus of DUHS from November 2019 to May 2021. All females aged above 40 years undergoing first time mammogram (screening or diagnostic) were enrolled. Of 639 included participants, 321 were included in paracetamol and 318 in placebo group. Patients in both the groups took medication orally which was customized by the Dow Pharmacy. The degree of pain felt during the mammography procedure was the outcome variable that was measured using Visual Analogue Scale.

**Results:**

The overall pain was found in 506 (79.19%) women. Pain was significantly higher in women who were in placebo group as compared to patients who were in paracetamol group, i.e., 280 (55.3%) and 226 (44.7%) (p-value <0.001). After adjustment of other covariates, the odds of pain was 3.64 times significantly higher in women who were in placebo group than that of women in paracetamol group (OR 3.64, 95% CI 2.31-5.74). Moreover, >25kg/m^2^ BMI was 2.84 times, 22.6-25 kg/m^2^ BMI was 2.29 times, nulligravida was 3.56 times, menopausal status was 2.23 times, pre-menopausal status was 4.51 times, and family history of breast cancer was 2.33 times significantly more likely to have pain. No post-trial complications were observed in both the groups.

**Conclusion:**

The use of paracetamol prior to the mammography procedure was found to be an effective intervention to reduce the pain among women.

***Clinical Trials:*** Identifier: NCT04381104.

## INTRODUCTION

Mammography is considered the fundamental screening tool for healthy women and an integral diagnostic procedure in women with both asymptomatic and symptomatic breast cancer.^1^,^2^ It provides soft tissue imaging by using low dose radiation. According to Global Cancer Project (GLOBOCAN), breast cancer is the most common malignancy in women, nearly 25.1% of all cancers which further emphasizes on the need of early detection, diagnosis and treatment.^3^,^4^ The lack of these actions lead to advancement in disease and high mortality rate in Pakistan.^5^ About 2.1 million women are affected per year.^6^ In Pakistan, the ratio of breast cancer is higher than rest of the Asian countries.^7^ It seems that most of the females refrain from mammography due to fear of pain and compression during this procedure.^8^,^9^ Published papers showed that certain interventions like analgesics, compression paddle/self-compression technique, radiolucent cushion, music therapy, lignocaine gel have been used to cope up the breast pain in women undergoing mammography.^10^-^12^

The goal is to improve the compliance of mammography screening and reduction of pain. Furthermore, earlier it was reported that anxiety, false perception, or fear causes the thought of discomfort while performing mammography.^13^ The rationale of this study is that the use of placebo and analgesics have been a focus for researches in developed countries pertaining to its benefits in improving the diagnostic health system. However, little to no attention is given to it in developing countries like Pakistan. In our study, paracetamol is used as a possible intervention as it is the safest and easily available premedication for reducing pain while performing mammography with standard compression protocol which is necessary to obtain a good quality image

## METHODS

This randomized double-blind placebo-controlled trial was conducted at Dow Institute of Radiology Ojha and LEJ Campus from November 2019 to May 2021 after taking Ethical Approval (IRB #: 1391/DUHS/approval/2019*)*. Furthermore, the trial was registered in Clinical Trial Registry (ClinicalTrials.gov Identifier: NCT04381104). The purpose of study was explained, and informed consent was also obtained from all eligible study participants. All female patients above 40 years of age, undergoing mammography for the first time either for screening/diagnostic purposes were enrolled. However, women who required supplementary projections, post-operative cases, tender breast, refused trial medication, or with prior history of paracetamol reaction were excluded. Estimation of sample size was done by Open Epi Info sample size calculator using following parameters; two-sided significance level (1-alpha) 95, power 81.3%, ratio of sample size one, percent of unexposed with outcome 45%, and percent of exposed with outcome 34%.^13^

The final sample size came out to be 639, i.e., 321 in Group-A (paracetamol) and 321 in Group-B (placebo). However, due to incomplete mammography procedure in three patients, 318 patients were included in Group-B. While placing appointment, all eligible participants were instructed to avoid any pain medication 24 hours before the examination. Patients were randomly divided into two groups (A): Paracetamol, (B): Placebo. The Pharmaceutical department of Dow Hospital, Karachi assisted in making customized capsules of each to provide a consistent appearance. Principal investigator unwrapped both the medicines, i.e., paracetamol and placebo, labelled them as “A” and “B” and put them into two similar envelopes.

Envelope distribution was performed with the help of randomized number list generated by applying formulation method in Microsoft Excel 2016. Alternatively, each patient received an envelope A or B having two capsules of 500mg paracetamol, or two capsules of placebo one hour prior to the procedure. Two standard views cranio-caudal and Medio-lateral-oblique projections were performed by using “Hologic Selenia 2-D Digital Mammography” and “Siemens Mammomat” equipment. Information regarding the demographic characteristics and a detailed clinical history was performed. Analgesic i.e., Paracetamol (Acetaminophen) which is widely used as a pain reducing medication with fewer or rare side-effects was used as medication.

Pain was defined as an uncomfortable and intolerable sensation such as twinge, pricking, throbbing, or aching during the mammography examination. Visual Analogue Scale (VAS) was used to assess the pain. The following cut points on VAS recommended: no pain (0), mild pain (1-3), moderate pain (4-6), and severe pain (7-10). Statistical analysis for Social Sciences (SPSS) version 24 was used for the purpose of statistical analysis. Descriptive statistics were explored using mean±SD and median (IQR) for quantitative and frequencies and percentages for qualitative variables. Inferential statistics were explored using Independent t-test, Mann-Whitney U test, and Chi-Square test. The p-value of ≤0.05 was considered as significant. Furthermore, binary logistic regression was also applied. All those variables found significant in chi-square were included in the binary logistic regression. Both univariable and multivariable analysis were applied.

## RESULTS

The 639 participants, the mean age was 48.01 ±9.95 years. There were 431 (69.6%) women with multigravida while multiparity was observed in 385 (62.2%) women. Urban residence was observed in 455 (71.2%) women. Married women were predominantly higher, i.e., 619 (96.9%). More than equal to intermediate education was observed in majority 277 (43.3%) women followed by less than equal to matric 218 (34.1%), while 144 (22.5%) were illiterate.

There were 133 (20.8%) women who reported no pain, 378 (59.2%) reported mild, 111 (17.4%) reported moderate, and 17 (2.7%) reported severe pain. The overall pain was found in 506 (79.19%) women. A significant association of pain frequency was observed with BMI (p-value <0.001), gravida (p-*value* <0.001), residence (p-*value* 0.021), menstrual status (p-*value* <0.001), familial history of breast cancer (p-*value* 0.006), and reason of test (p-*value* .002). (Table-I & II)

**Table-I:** Comparison of pain in mammography with baseline characteristics.

Pain				

	Overall (n=639)	Yes (n=506)	No (n=133)	p-value

	n	n (%)	n (%)	
Age, years [mean ±SD]	48.01 ±9.95	48.00 ±10.15	48.05 ±9.16	0.960
<40	199	159 (31.4)	40 (30.1)	0.546
40-55	297	230 (45.5)	67 (50.4)	
>55	143	117 (23.1)	26 (19.5)	
Weight, kg [mean ±SD]	63.02 ±11.99	64.11 ±12.06	58.88 ±10.75	<0.001
Height, m [mean ±SD]	1.61 ±0.06	1.61 ±0.06	1.62 ±0.06	0.998
BMI, kg/m2 [mean ±SD]	24.24 ±4.76	24.67 ±4.85	22.62 ±4.01	<0.001
<18.5	76	51 (10.1)	25 (18.8)	<0.001
18.5-22.5	184	128 (25.3)	56 (42.1)	
22.6-25	139	120 (23.7)	19 (14.3)	
>25	240	207 (40.9)	33 (24.8)	
Age at time of marriage, years (n=619) [mean ±SD]	20.65 ±4.29	20.67 ±4.22	20.55 ±4.56	0.779
≤25	554	444 (90.8)	110 (84.6)	0.041
>25	65	45 (9.2)	20 (15.4)	
Gravida (n=619) [median (IQR)]	3 (2-5)	3 (2-4)	4 (3-5)	<0.001
Nulligravida	70	61 (12.5)	9 (6.9)	<0.001
Primigravida	24	22 (4.5)	2 (1.5)	
Multigravida	431	339 (69.3)	92 (70.8)	
Grand Multigravida	94	67 (13.7)	27 (20.8)	
Parity (n=619) [median (IQR)]	3 (1-4)	3 (1-4)	3 (2-5)	0.029
Nulliparous	114	98 (20.0)	16 (12.3)	0.130
Primiparous	47	35 (7.2)	12 (9.2)	
Multiparous	385	305 (62.4)	80 (61.5)	
Grand Multiparous	73	51 (10.4)	22 (16.9)
***Residence***				
Rural	184	135 (26.7)	49 (36.8)	0.021
Urban	455	371 (73.3)	84 (63.2)
***Education***				
Illiterate	144	106 (20.9)	38 (28.6)	0.159
Less than equal to matric	218	178 (35.2)	40 (30.1)	
More than equal to intermediate	277	222 (43.9)	55 (41.4)
***Marital Status***				
Unmarried	20	17 (3.4)	3 (2.3)	0.515
Married	619	489 (96.6)	130 (97.7)
***Occupation***				
Working women	572	448 (88.5)	124 (93.2)	0.116
Non-working women	67	58 (11.5)	9 (6.8)	

Independent t-test applied, Mann-Whitney U test applied, Chi-square test applied.

**Table-II:** Comparison of pain in mammography with clinical characteristics.

		Pain		

	Overall (n=639)	Yes (n=506)	No (n=133)	p-value

	n	n (%)	n (%)	
***Previous lactation status***				
Positive	477	367 (72.5)	110 (82.7)	0.016
Negative	162	139 (27.5)	23 (17.3)
***Menstrual Status***				
Normal	329	232 (45.8)	97 (72.9)	<0.001
Peri-menopausal	76	71 (14.0)	5 (3.8)	
Menopause	234	203 (40.1)	31 (23.3)
***Palpable Lump***				
Yes	278	217 (42.9)	61 (45.9)	0.537
No	361	289 (57.1)	72 (54.1)
***Family History of Breast Cancer***				
Yes	114	101 (20.0)	13 (9.8)	0.006
No	525	405 (80.0)	120 (90.2)
***Current Hormone Therapy***				
Yes	23	21 (4.2)	2 (1.5)	0.145
No	616	485 (95.8)	131 (98.5)
***Comorbidities***				
HTN	141	109 (21.5)	32 (24.1)	0.138
Diabetes	48	39 (7.7)	9 (6.8)	
HTN and DM both	45	30 (5.9)	15 (11.3)	
None	405	328 (64.8)	77 (57.9)
***Prior Test Knowledge***				
Yes	65	52 (10.3)	13 (9.8)	0.865
No	574	454 (89.7)	120 (90.2)
***Allergy to medicine***				
Yes	6	6 (1.2)	0 (0)	0.207
No	633	500 (98.8)	133 (100)
***Reason of Test***				
Screening	267	227 (44.9)	40 (30.1)	0.002
Diagnostic	372	279 (55.1)	93 (69.9)	

Chi-square test applied, p-value <0.05 considered as significant.

A significant association of frequency and severity of pain was observed in between group (p-value <0.001). The findings of the multivariate analysis revealed that the odds of pain was 3.64 times significantly higher in women who were in placebo group than that of women in paracetamol group (aOR 3.64, 95% CI 2.31-5.74). The odds of pain was 2.84 times significantly higher in women with >25 kg/m^2^ BMI and 2.29 times higher in women with 22.6-25 kg/m^2^ BMI than that of women with <18.5 kg/m^2^ BMI, i.e., (aOR 2.84, 95% CI 1.43-5.62) and (aOR 2.29, 95% CI 1.07-4.91). The odds of pain was 2.2 times significantly higher in women with ≤25 years of age at the time of marriage than that of women with >25 years of age at the time of marriage (aOR 2.20, 95% CI 1.14-4.26). The odds of pain was 3.56 times significantly higher in nulligravida women than that of women with grand-multigravida (aOR 3.56, 95% CI 1.39-9.09). The odds of pain was 2.23 times significantly higher in menopausal women and 4.51 times significantly higher in peri-menopausal women than that of women with normal menstrual status, i.e., (aOR 2.23, 95% CI 1.35-3.68) and (aOR 4.51, 95% CI 1.67-12.18). The odds of pain was 2.33 times significantly higher in women with family history of breast cancer than that of women without family history of breast cancer (aOR 2.33, 95% CI 1.19-4.57). (Table-III, IV)

**Table-III:** Comparison of pain with respect to groups.

	Pain			p-value

	Overall (n=639)	Yes (n=506)	No (n=133)	

	n	n (%)	n (%)	
Medicine				
Paracetamol	321	226 (44.7)	95 (71.4)	<0.001
Placebo	318	280 (55.3)	38 (28.6)	

Chi-square test applied, p-value <0.05 considered significant.

**Table-IV:** Regression analysis of variables associated with pain in mammography.

	OR (95% CI)	p-value	aOR (95% CI)	p-value
***Group***				
Placebo	3.09 (2.05-4.69)	<0.001	3.64 (2.31-5.74)	<0.001
Paracetamol	Ref		Ref	
***BMI, kg/m^2^***				
>25	3.07 (1.68-5.62)	<0.001	2.84 (1.43-5.62)	0.003
22.6-25	3.09 (1.57-6.12)	0.001	2.29 (1.07-4.91)	0.032
18.5-22.5	1.12 (0.63-1.98)	0.697	1.18 (0.61-2.27)	0.621
<18.5	Ref		Ref	
***Age at time of marriage, years (n=621)***				
≤25	1.79 (1.02-3.16)	0.043	2.20 (1.14-4.26)	0.019
>25	Ref		Ref	
***Gravida***				
Nulligravida	2.73 (1.19-6.27)	0.018	3.56 (1.39-9.09)	0.008
Primigravida	4.43 (0.97-20.17)	0.054	3.81 (0.73-19.59)	0.113
Multigravida	1.48 (0.89-2.45)	0.123	1.50 (0.84-2.68)	0.167
Grand Multigravida	Ref		Ref	
***Residence***				
Rural	0.62 (0.42-0.93)	0.022	0.74 (0.47-1.19)	0.224
Urban	Ref		Ref	
***Menstrual status***				
Menopause	2.74 (1.75-15.16)	<0.001	2.23 (1.35-3.68)	0.002
Peri-menopausal	5.94 (2.33-15.16)	<0.001	4.51 (1.67-12.18)	0.003
Normal	Ref		Ref	
***Family History of Breast Cancer***				
Yes	2.30 (1.25-4.25)	0.008	2.33 (1.19-4.57)	0.014
No	Ref		Ref	
***Reason of test***				
Screening	0.37 (0.24-0.56)	<0.001	1.43 (0.89-2.31)	0.135
Diagnostic	0.24 (0.14-0.42)	<0.001	Ref	

OR: Odds Ratio, AOR: Adjusted Odds Ratio, CI: Confidence Interval.

## DISCUSSION

Recent studies have shown that 91% of the women undergoing mammography procedures feel low to moderate pain.^14^ The burden of feeling discomfort, and anxiety resulted in dissatisfaction and delay in mammography.^15^ As medical practitoners, it is our responsibility to allow control over the procedure, which may increase their compliance to the screening programs.

This study investigated the effect of paracetamol and placebo in reducing discomfort during mammography. As the patients were not aware of the medicine composition, it helped conclude if the pain is not only due to perceived mindsets. The study reported that pain killers had an effective role during the mammography procedure. Similar findings were reported in previous studies as well.^16^-^20^ Obesity was a crucial variable observed during our study. A correlation between BMI and compression tolerance threshold was seen. A recent study reported similar findings stating that the tolerance in the patient to tolerate the pain decreases as the body weight increases.^18^ Moshina et al., however, reported that the BMI and pain tolerance has no effect on each other.^21^

Considering the ages of our patients, we observed that the intensity of pain felt in menopause women was higher than the younger probably due to the increase in age and intolerance. This, however, contradicted with a previous study that proved that being in menopause as well as the few variables like coffee intake, education level and age do not have any correlation with pain.^18^-^21^ The mammography procedure hurt more in women who got married before the age of 25 years and are multigravida. Furthermore, our results showed odds of pain was significantly higher in women with familial history of breast cancer. This further coincides with two studies conducted previously.^21^,^22^ This proves that because they had a previous insight on how the procedure goes about and the circumstances, they tried to avoid going into the journey.

Urban population felt more pain in contrast to rural which is likely due to impact of prior misconception that they hear from relatives, friends, and family. A study in China proved that fatalism is higher in less educated women which could lead to perceived pain.^24^

### Limitations:

In the current study, no control group were enrolled. Moreover, due to COVID-19 pandemic, low patient flow led to delayed data collection. In this study, anxiety related psychological scoring and menstrual phases were not considered.

Despite these few limitations, the large sample size of this study makes it an important one. Moreover, conducted in a large public sector radiology institute of Pakistan, this study has reported findings from patients belonging to diverse areas and languages. Further studies are recommended that include the pain threshold in follow-up cases. A comparison study of all the manuvors like gels, painkillers, and music therapy can be used to allow proper investigation of the efficiency of each of them.

## CONCLUSION

The use of paracetamol prior to mammography procedure was found to be an effective intervention to reduce the pain.

### Authors’ Contribution:

**AR, BR & SOA:** Conceived, designed and did statistical analysis & editing of manuscript, is responsible for integrity of research.

**AR, BR, NN & NA:** Did data collection and manuscript writing. All authors gave final approval of manuscript

**AR** checked for the accuracy and integrity of the manuscript.
